# Effect of cooking food in iron-containing cookware on increase in blood hemoglobin level and iron content of the food: A systematic review

**DOI:** 10.3126/nje.v11i2.36682

**Published:** 2021-06-30

**Authors:** Shally Sharma, Ritika Khandelwal, Kapil Yadav, Gomathi Ramaswamy, Kashish Vohra

**Affiliations:** 1 Department of Food and Nutrition, Department of Food and Nutrition, Lady Irwin College, University of Delhi, New Delhi, India; 2,3 Centre for Community Medicine, All India Institute of Medical Sciences, New Delhi, India; 4 Department of Community Medicine and Family Medicine, All India Institute of Medical Sciences, Bibinagar, Telangana, India; 5 Centre for Community Medicine, All India Institute of Medical Sciences, New Delhi, India

**Keywords:** Anemia, Iron deficiency anemia, iron ingots, Iron pots, hemoglobin

## Abstract

In developing countries there is a need for simple and cost-effective strategies to reduce the prevalence of iron deficiency anemia. The objective of the current systematic review is to summarize how cooking food in iron pots or iron ingots can increase the blood hemoglobin level and iron content of the food. Literature search was conducted using databases namely PubMed, Google Scholar, Medline-Ovid, IndMed, Cochrane library, World Health Organization bulletin and by cross-referencing articles. Thirteen researches were found to be suitable for inclusion in this systematic review. Four studies reported significant increase in blood hemoglobin levels while others reported only a minor increase. Significant improvement in amount of iron in food and iron bioavailability was also observed when food was cooked using iron pot or ingots. This can be used as a strategy for reduction of iron deficiency anemia. However, more research is required to understand the efficacy of this approach.

## Introduction

Anemia, characterized by ‘low blood-hemoglobin concentration’, is a key public health concern prevalent among low, middle as well as high-income countries, worldwide [1]. Almost 1.62 billion people are affected from anemia, which approximates to 24.8% of the world’s population [2]. According to the Global Database on anemia, it is most frequent among children of preschool-age (47.4%), followed by pregnant (41.8%) and non-pregnant (30.2%) women [2]. There are various underlying causes of anemia, of which the most significant contributor is iron deficiency anemia (IDA). It is supposed that fifty percent of anemia instances are attributed to deficiency of iron [2]. As per National Family Health Survey-4 (2015-2016) India, the prevalence of IDA is more common in the rural setup as compared to the urban areas [3].

Iron deficiency occurs predominantly due to malnutrition and it is commonly observed in a section of people with rapid rate of growth [4]. IDA has its adverse effects on cognitive and motor development, which leads to low productivity and fatigue [5]. Thus, it is important to address this public health problem.

A number of stakeholders or sectors namely health, agriculture, industry, education, and communication can contribute to prevent anemia among general population. There are various preventive strategies for IDA which include dietary improvement, food fortification, biofortification, iron supplementation, nutrition counselling, integration with other micronutrient control programme and antiparasitic treatment [6]. The reduction of anemia (IDA) is a global challenge which is more profound in developing countries. In India, the Ministry of Family and Health Welfare (MoHFW), revamped the existing National Iron Plus Initiative (NIPI) in the year 2018 as Integrated National Plus Initiative – Anemia Mukt Bharat to combat anemia [7]. The program focusses on providing iron folic acid supplementation along with deworming to improve the hemoglobin status among various age groups of both genders, i.e., children (6-59 months, 5-9 years), adolescent (10-19 years), pregnant and lactating women and females of reproductive age (20-49 years) [9].

Despite the national and international anemia prevention programmes, the burden of IDA still remains high. Iron supplementation despite being the primary strategy to combat IDA in India; however, there is no substantial abatement in the anemia prevalence within the country. Moreover, effectiveness of iron supplementation largely depends on the delivery of supply chain system and compliance of the target recipients [8]. In addition, supplementing with iron can cause unwanted side-effects including gastro intestinal symptoms like nausea, vomiting, pain abdomen and constipation [9]. Staple food fortification is alos considered as one of the major economical intervention [6]. However, this intervention is also largely dependent on supply chain system and involvement of large- and small-scale manufacturers of rice to comply with iron fortification at production site [10]. Therefore, the daily requirement of dietary iron intake is difficult to meet. The main etiological factor for nutritional anemia is the low dietary iron intake and it’s low availability [11,12]. In developing countries, easy accessibility of iron and vitamin C-rich food remains uncertain, however cereals and beverages like tea and coffee which contains polyphenols, phytates and tannins, are often dietary staples [13].

One-fifth of the global population is affected by IDA, in spite of the availability of several approaches that addresses the same [14]. An earlier systematic review by Geerlings et al reported that use of iron pot for preparing food may help to overcome the iron deficiency anemia in developing countries [15]. In India an action-oriented and cost-effective intervention is needed to reduce anemia especially among the vulnerable population, with minimal requirement for active compliance of individuals. Use of iron pot or ingot for cooking is considered as one such intervention that targets the whole family instead of individual level intervention. They cultural acceptability, given the history of usage of iron utensils for cooking in India. Herein, the motive of this systematic review of scientific literature was to study the impact of using iron-containing cookware (iron pot and ingot) for cooking on hemoglobin level amongst individuals and on the iron content of the food.

## Methodology

### Literature Searches

A systematic literature search of randomized and non-randomized trials was performed by two authors using scientific databases such as PubMed, Google Scholar, Medline-Ovid, IndMed, Cochrane library and bulletin of World Health Organization. The search strategy comprised of free texts and Medical Subject Headings (MeSH) such as - ‘iron pots’, ‘iron utensils’, ‘iron vessels’, ‘iron ingot’, ‘iron’ AND ‘cooking’, ‘cooking and eating utensils’ AND ‘iron deficiency anemia’; ‘prevention of iron deficiency’ and ‘anemia’. Various combinations of these keywords were used for literature search. The bibliographies of the selected articles were also referred to find further relevant studies.

### Inclusion and Exclusion criteria

Randomized trials and experimental studies assessing the outcomes of cooking in iron-containing cookware on blood hemoglobin status of individuals and on the total amount of iron in food items were included. The original research articles with accessibility to full text and published in English language, from 1991 till December 2020, were included.

Whereas, animal studies, review articles, conference proceedings, commentaries, and editorial opinions were excluded. No restrictions were given for the study setting, study duration, study population or outcome assessment technique.

We have used PRISMA (Preferred Reporting Items for Systematic Reviews and Meta-Analyses) diagram to represent flow of data in all phases of this systematic review[16].

### Data Extraction

The title and abstracts of selected articles were reviewed by two independent authors for suitability. The articles which were identified as relevant by both the authors were considered for the final review. Disagreement between the authors were sorted out after discussion or opinion from the third author was obtained for the inclusion of the articles. Extracted data included: sample size, study design, location, period, intervention done and findings of the study.

Table 1 summarizes findings of included studies which were mainly performed at community settings, household or laboratory.

### Methodology Quality Assessment

Two reviewers individualistically completed methodology quality assessment of involved randomized control trials using “Cochrane Collaboration’s tool for assessing risk of bias in randomised trials” [17]. This tool assessed quality of the included studies on six criterias of bias; selection, detection, performance, reporting, attrition, and other bias. No methodology quality assessment was done for laboratory studies.

### Outcome measures

- The primary outcome measure was the blood hemoglobin level to evaluate the efficacy of using iron-containing cookware for cooking food. Other hematological parameters (like serum ferritin) were also taken into account, if available.
- The secondary outcome of interest was iron content of the cooked food.

## Results

**Figure 1.**
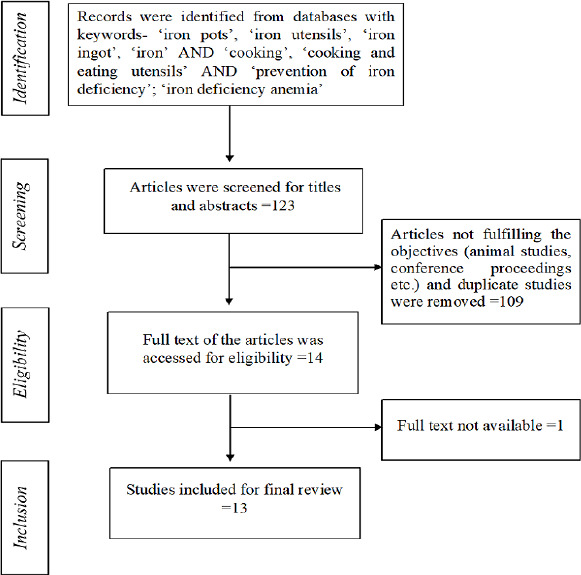
– Figure 1- PRISMA flow diagram which included database search using keywords, title, abstract screening and full text

**Figure 2.**
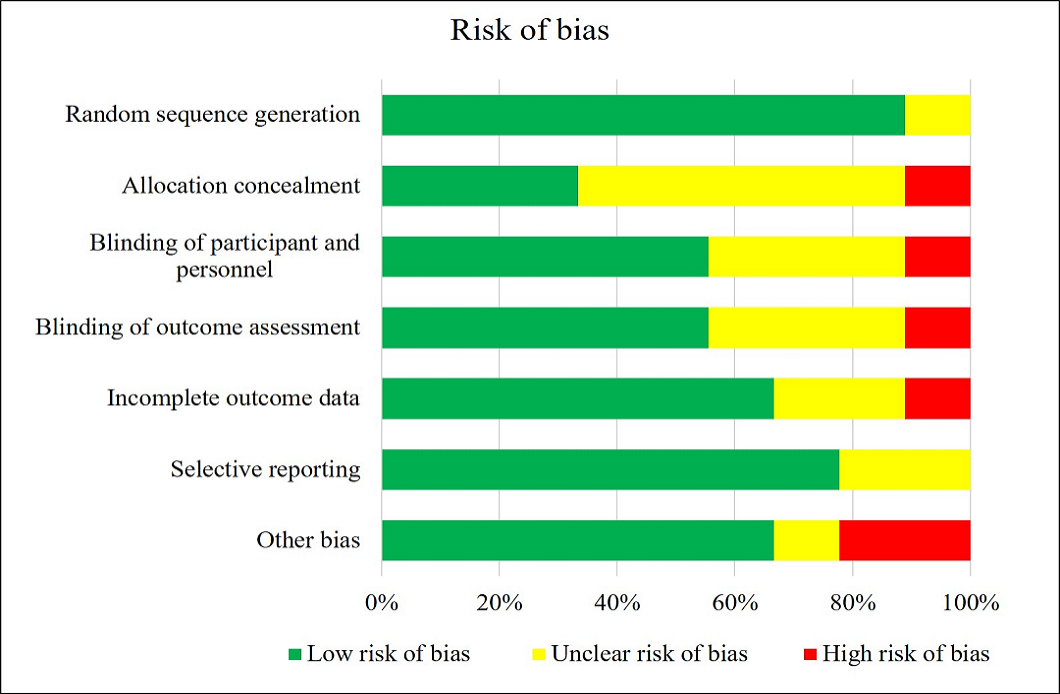
Risk of bias as percentage for included randomized trials

**Figure 3.**
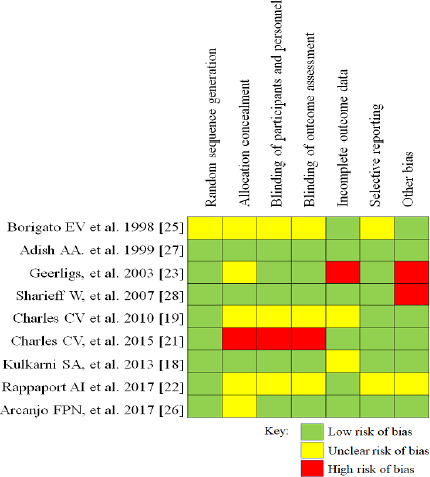
Risk of bias summary

The initial literature search resulted in total 123 articles, from which 13 articles were finally included for the systematic review [20-32]. The PRISMA flow diagram of selection and exclusion of studies is depicted in Figure 1. All included studies except one were conducted outside India [20]. Four studies were reported from Cambodia [19-22], two studies from Malawi [23-24], two from Brazil [25-26], one study each from Ethiopia [27], Benin [28], China [29] and USA [30]. Randomized, non-randomized or basic experimental trials on iron content of food items, published between 1991 to 2020 were included. Nine studies were randomized controlled studies which considered change in hemoglobin level as the proxy parameter to assess the change in iron status [18,19,21-23,25-28]. Of these nine articles, four performed laboratory testing to check for the change in quantity of iron in food cooked using iron-containing cookware [20,24,29,30]. Methodological quality assessment was done for nine randomized control studies (Figure 2 & 3). Overall quality of studies was high, nevertheless, allocation concealment bias was highest among all the biases. Least amount of bias was observed in random allocation generation. However, unclear risk of bias was found among few articles for some parameters.

The sample size varied from 27 to 407 in the reviewed articles [18,19,21-23,25-27]. Of the 13 studies included, 9 were conducted among human subjects and four were purely laboratory-based studies to assess the change in iron content of the food. The total sample size for the nine studies were 2096 subjects. Across all the studies the participants were children, adolescents and women. In the four-laboratory based studies food was cooked in different pots or with iron ingots and difference in iron content was measured [20,24,29,30].

Method used for hemoglobin estimation was Hemocue (point of care testing using digital hemoglobinometer) in six of the studies [21-23,26-28], hemoglobinometer in one study [25], one study did not mention the method of estimation [19], one study used plasma optical emission spectroscopy [20] and for rest of the studies atomic absorption spectrophotometry method was used [24,26,29,30].

### Effect on hemoglobin

The duration of studies varied from 16 weeks to 12 months. Four studies reported a significant increase in hemoglobin levels [18,21,25,27] while others reported only a slight increase [21,24,25,28,30]. The highest change in hemoglobin was observed in the study by Adish et al. in 1993, where an increase of 1.7g/dl in hemoglobin level from baseline to endpoint (12 months) was observed (95% CI 1.1-1.6g/dl); followed by a change of 0.8g/dl in the study on preschoolers, in which iron pot was used for 4 [18,27]. Minimum change in blood hemoglobin levels was seen, i.e. 0.03g/dl, post 16 weeks of intervention, for another group of preschoolers [26]. No significant change in mean blood hemoglobin levels was observed in children below 12 years of age during follow-up. For children >12 years of age, mean hemoglobin increased significantly among consistent iron pot users after 6-weeks and 20-weeks of follow-up (95% CI: 0.86-12.74; p=0.04) [23]. This study was conducted in high malaria prevalence region of Malawi. No difference in blood hemoglobin levels were reported across both the genders on using iron-containing cookware (iron pot or ingot[16,23,24,25,27,28].

### Reduction in iron deficiency anemia prevalence

Sharieff et al showed decline in prevalence of IDA between both the groups (cast iron pot users and blue steel pot users) post-intervention but this reduction was not statistically significant [28]. In another cluster randomized clinical trial, schools were provided with iron pots for meal preparation at school for the participants (preschoolers). In this group, prevalence of anemia decreased from 12.2% to 8.5% after 16-weeks of intervention. Preschoolers who were anemic at the baseline, were found to be non-anemic by the end of intervention (p< 0.001) [26]. Similar trend was also observed in a study which showed decrease in anemia prevalence from starting (73%) to study endpoint (54%), i.e., by 4 months of supplementing snacks (made using cauliflower greens with either bengal gram flour, or soyabean, or cowpea and laddoos made from garden cress and sesame seeds), cooked in iron pot (p<0.05) [18].

Decrease in anemia prevalence was also observed in the group that used iron ingot for cooking food [21]. Overall, anemia reduced by 46% in intervention group where participants were using the ingot daily for 12 months. Hemoglobin concentrations were >1.18 g/dL (95% CI: 9.1-14.6; p<0.001) amongst iron ingot intervention group women when compared to control group [21]. Contrarily, another randomized control trial did not observe any signiﬁcant difference in mean blood hemoglobin between iron-ingot or iron supplement group compared to controls after 12-months [22].

### Change in iron content of cooked food

Experimental studies demonstrated change in total iron quantity of food items cooked using iron pot or iron ingot. Some of the studies reflected a significant rise in total content of iron in the food preparations post cooking in iron pots and iron ingots [13,18,20,27,29]. Highest increase in iron content was observed in meat and vegetable preparations when compared to legumes [27]. Amount of iron doubled in meat and vegetables and increased by 1.5 times in legumes when cooked in iron pots, as compared with other two pots. Iron content of the pea pastes was 3.3 times higher (21.4±1.0 mg) when prepared in iron pot in comparison to peas prepared in clay pot (p<0.05) [29].

Lemon water made using iron ingot had more iron content (p<0.001) than the controls made without the ingot [20]. Comparison of apple and spaghetti sauces prepared in both iron and non-iron pots, showed a significant increase in the content of iron in apple sauce prepared in iron pot (p>0.05) [30].

### Acceptability of iron pot

Participants were also enquired regardingacceptability of iron pot for cooking purpose [23,24,31]. Participants were provided with either iron or aluminum pots, some of the participants preferred aluminum pots and not iron pot because former were lighter in weight, smaller in size and had flat base [31]. Notably, it was observed that the number of meals being cooked in the aluminum pot decreased significantly over 17-weeks, however, no significant change was found in households using iron pot for cooking (p=0.001). No change in mean acceptability score for aluminum pot was observed over time but there was a significant decline in acceptability score for iron pot in 17-weeks, i.e., from 13.7 to 11.4 (range 1-20) (p=0.001) [31]. Also, the percentage of people considering iron pot of good quality decreased significantly by the end of the study from 63% to 40% (p=0.04). The major reason for this decline in acceptability was the physical attributes of the pot, i.e., rusting, heaviness of the pot and three leg pot (which were distributed by a researcher). The primary reasons for acceptability of iron pots mentioned by the participants were that it heats up early, saving time and fuel; food was being easily prepared, and the durability of the pot [31]. On the other hand, iron pots were being replaced by aluminum pots because the latter were cheap, light weight, rust-resistant and readily available [32].

### Acceptability of iron ingot

Compliance was also seen regarding the use of iron ingot for cooking. Initially, participants were advised regarding maintaining dryness and cleanliness of iron ingot after cooking, to avert rust formation. The compliance of iron ingot throughout the study period was high, as 94 percent of participants were daily using the iron ingot [21].

## Discussion

This systematic review identified change in blood hemoglobin levels, reduction in iron deficiency anemia prevalence and change in quantity of iron in food after cooking in iron pot and ingot for different age groups. In all the included trials, iron pots and iron ingots were provided to different populations, to consume food prepared in iron pots and using ingots [18-19,21-23,25-28].

### Change in hemoglobin levels

Most studies have observed an increasing trend in hemoglobin levels in all population groups that used iron pots, but only four of them have reported a significant change [18,21,25,27]. Rest of the studies reported small and non-significant changes in hemoglobin levels [20,22,23,26,28]. In children who were infected with malaria, there was an insignificant rise in mean blood hemoglobin levels. This might be due to hemolysis of erythrocytes induced by malarial parasites, which affects the recovery of children from anemia. Thus, in malaria-endemic countries, the disease could have influenced the outcome assessment [23]. Furthermore, in women (18-49 years) from Cambodia where the prevalence of iron deficiency was low, iron ingot did not increase hemoglobin concentrations at 6 or 12 months [22].

### Impact on cooked food

It is clear from the laboratory studies that iron content increased more in cooking meat in the iron pots than vegetables and legumes. The possible reason could be the presence of non-heme iron in legumes and vegetables which has low degree of absorption than heme iron [27]. The included studies have observed both significant as well as non-significant decrease in the prevalence of iron deficiency anemia. One study reported total elimination of IDA from baseline at study end point (16 weeks) [26]. It was observed that the soups cooked using iron ingot have significantly more amount of iron than those cooked in without it. The highest content of iron was noted for water boiled with lemon juice using iron ingot. It is clear that acidity of food is essential for making the most of iron leaching and enhancing its absorption as 76.5% of daily iron needs were met when 1 liter lemon water made using the ingot was consumed [20].

### Benefit of follow-up and counselling

In absence of follow-up, there was a slight increase in the participants’ hemoglobin level, whereas, much improvement was observed in the follow-up category [19]. This suggests that proper follow-up of participants provided with iron pots ensures more regular usage and better outcome as compared to those without follow-up. At first, some unwillingness was there for using iron ingot but after counselling, the participants were comfortable in regularly using the ingot [21].

### Choice of pot and acceptability

The benefit of using iron pot rest on multiple factors such as prevalence of malaria in population, hookworm infestation, satisfaction and comfort of the participants in using the iron pots for cooking. Hence, it is crucial to spread public awareness for the benefits of using iron pots or ingot and improve its acceptability. It is also necessary to improve iron pot and iron ingot availability for wider usage by the general population. The iron pots that were distributed among the Malawian population were brought from outside the village and were much larger in size than aluminum pot, which was one of the reasons that iron pots were lesser acceptable by the households [31]. Buying smaller size pots from the near-by locations and availability of attractive pots could have helped in combating this problem. Also, rusting (iron oxidation) was one of the reason for non-compliance which is an inherent property of iron pots and can be prevented by seasoning the pot with cooking oil [31]. Though, iron pots are more culturally acceptable in rural setup as they have been used since old times, yet they are underutilized in India. Iron pots are now being replaced by aluminum or stainless-steel pots which are lighter in weight and durable (rust-free).

### Supplementation of iron: Iron ingot

Among the population who are not willing to use iron pots, iron ingots are potential option. They are light-weighted, economical, have a longer lifespan (>5 years), and can be used in any cooking pots, irrespective of manufacturing material. No alteration in color or taste of food was found upon cooking food using iron ingot, which could have motivated people to use it throughout the study. Also, no adverse effects were reported by any participant or research team on utilization of iron ingot [19,21,22]. In a Cambodian study, the iron ingot were distributed among participants which reveal high compliance rate (94%) to use the ingot daily. Compliance for iron ingot was found to be higher than iron pot, as also observed by Alves et al. in their review, but only limited studies are available on compliance to draw any conclusion [33].

Iron deficiency is among the major reasons for death in India touching upon more than 50% of its population [34]. It unfavorably affects the motor development, coordination and language development in infants; cognitive performance, growth and behavior of children; resulted in lower immunity and higher morbidity from infections among all age groups [35-38]. Therefore, it is imperative to eliminate the IDA from the population especially among the preschoolers and women of reproductive age in whom this deficiency is more prevalent. Regular use of utensils or cooking equipment made from iron can help in alleviating this problem. Similar results have been discussed by Alves et al in their systemic review stating the use of iron-containing cookware and iron ingots, with fair compliance, as a strategy to lessen IDA among populations, especially among children [33]. Cooking in iron pots and using ingots have certain benefits which are being overlooked such as it saves time and fuel and can also be used for longer time as they do not get damaged easily. In addition, intake of vitamin C along with food prepared using iron pot and ingot, promotes iron absorption as former acts as a promoting ligand for absorption.

### Expert opinion

Clinical trials and experimental studies aid in understanding the results of treatment interventions. Low cost effective interventions are needed in developing countries to reduce mortality rate and in ensuring good health, free of diseases and deficiencies. Currently many population groups from different sections of the society, throughout the world, are suffering from IDA. This public health problem is posing risk to life of many people, especially preschoolers and women of reproductive age, and needs to be treated. As discussed in this review, cooking in iron pot has been found to be beneficial in improving the blood hemoglobin levels of people suffering from IDA. Using of iron pot and iron ingot for cooking food could be acceptable among the population as iron pot saves their time, firewood and money, making it economical for them. Also, the taste of food cooked using iron pots and with iron ingots is not altered and satisfactory, as observed in studies.

It is important that people should be educated about the use of pots and ingots, how frequently the pots should be changed and importance of this strategy for their health. Proper monitoring and reinforcement should be done at frequent intervals. While advising people to use iron pot and ingots, one should make sure that excess iron intake should also not be done, to avoid toxicity related complications. Other strategies should also be enforced along with using pot and ingot, like inclusion of heme iron and Vitamin C rich sources in diet, and avoiding intake of calcium rich sources with food containing iron as former inhibits the absorption of latter.

Millions of lives can get benefitted throughout the world by making this small alteration in their daily life. This can prove to be an advantageous approach while planning strategies to improve public health nutritional status.

## Limitation of the study

The major limitation in this study was the unavailability of ample studies in assessing the effect of using iron pot/utensil and iron ingot for cooking and prevention of iron deficiency. Also, some of the literature which was available had lesser sample size to draw strong conclusions and recommendations.

## Conclusion

It can be inferred that cooking food in iron pot escalates the levels of blood hemoglobin and iron content of the food, and thus reduces the incidences of iron deficiency anemia. The bioavailability of food containing heme iron increases more when cooked in iron pot than food having non-heme iron form. Also, the content of iron in the food was found to be increased by cooking acidic food with iron ingot. Very limited research trials are available on this topic that warrants a careful interpretation of results inferred and a considerable need of larger population-based studies and randomized controlled trials for better outcomes.

## Figures and Tables

**Table 1.** Summary of literature on the effect of cooking in iron containing cookware and using iron ingot on anemia status of the study participants

Author	Year	Country	Study design and duration	Participant Characteristics	Intervention	Findings
**Borigato EV, et al. [25]**	1998	Brazil	RCT^++^ 8 months	n= 45 Age= 4 months ^##^M:F-10:12 (Fe* pot) M:F-11:12 (Al^#^ pot)	Infants consumed food prepared in Fe pot or Al pot	Mean hemoglobin (+0.52g/l) in Fe pot significantly increased as compared to Al pot (-0.74g/dl)
**Adish AA, et al. [27]**	1999	Ethiopia	RCT 12 months	n= 407 Age= 2-5 years M:F-99:96 (Fe pot) M:F-106:106 (Al pot)	Three meals cooked in Al, clay & Fe pot. Consumption of food prepared in Fe & Al pots	More increase in crude Fe content in meat and vegetables than legumes Hb^+^ increased by 1.7g/dL from baseline (p=0.008), proportion of IDA** reduced from 57% to 13%
**Geerligs, et.al. [23]**	2003	Malawi	RCT 5 months	n= 322 Age: <12 yrs M:F-0.9 (Fe pot) M:F-1.1 (Al pot) Age: >12yrs M:F- 0.6 (Fe pot) M:F- 0.7 (Al pot)	Fe or Al cooking pot assigned for cooking	< 12 years: No increase in Hb, > 12 years: Significant Hb rise at 6 weeks (+0.36g/dl) & 20 weeks (+0.53g/dl) in consistent Fe pot users (p<0.05)
**Geerligs PP, et al. [24]**	2004	Malawi	Experimental laboratory trial		Three Malawian meals prepared in two Fe pots and a glass pot	Fe content improved from 3.15 μg to 147.32 μg/g in food, when cooked in Fe pot Continuous cooking further increased Fe content by 2.9 μg to 20.1 μg Fe/g.
**Sharieff W, et.al [28]**	2007	Benin, Africa	Cluster RCT 6 months	n=71 children, age= 6-24 months; n=92 adolescent girls, age=11-15 years; n= 131women, age=15-44 years	Two groups used cast Fe or blue steel pots and controls had Fe supplements	Insignificant differences in mean Hb or IDA among groups, SF^+++^ was higher in control group (p<0.0001)
**Charles CV, et al. [19]**	2010	Cambodia	RCT 6 months	n=189 women Age= >16y with haematocrit >30%	3 groups: control, received Fe fish with no follow up and received Fe fish with follow up	No significant change in Hb
**Charles CV, et al.** **[20]**	2011	Cambodia	Experimental study	-	4 groups: Controls: glass pot and Al pot; glass pot with Fe fish; and Al pot with Fe ingot	Use of iron ingot while making water and soup samples met more daily iron needs
**Charles CV, et al. [21]**	2015	Cambodia	RCT 12 months	n=310 pre- and post-menopausal women	3 groups: Fe ingot, Fe ingot plus nutrition education, and untreated control group	Mean Hb concentration varied by 1.18 g/dL across control and treatment group (p<0.001) IDA reduced by 46% in intervention group
**Kulkarni SA, et al. [18]**	2013	India	Randomised trial 4 months	n= 27 pre-schoolers, mean age 2.9±0.9y M:F- 12:15	Supplementation with a snack cooked in Fe pot, for 5 days/week	Hb improved from 10.1±1.6 to 10.9±1.7 g/dL (p<0.001) IDA reduced by 19%
**Rappaport AI, et al. [22]**	2017	Cambodia	RCT 12 months	n=327 women, mean age 32 years	3 groups: iron-ingot, iron-supplement (18 mg/d), and control group	Insignificant differences in mean Hb concentration among iron-supplement or ingot group compared with control
**Arcanjo FPN, et al. [26]**	2017	Brazil	Cluster RCT 16 weeks	n=175 preschoolers Age= < 59 months M:F- 52:41 (Fe pot) M:F- 48:34 (Al pot)	Consumption of food cooked in Fe pot or Al pot	No significant increase in Hb for non-IDA children, Hb (Fe vs. Al): +1.69g/dl vs. +1.10g/dl for anemic children No IDA in iron pot group
**Xing Q, et al. [29]**	2017	China	Experimental study	Panel of 12 trained tasters (4 males & 8 females)	Five pea samples soaked in deionized water, boiled in Fe pot & clay pot with variable iron content (0, 0.05, 0.50, 5.00 mg/L FeSO_4_) and freeze dried.	Pea pastes in Fe pot had 3.3 times higher Fe content (21.4±1.0 mg) than clay pot (p<0.05), more FeSO_4_ (wt/vol), more Fe content in pea
**Cheng YJ, et al. [30]**	1991	USA	Experimental study	-	Fe content compared in raw sauce samples, sauce prepared in Fe pot and non Fe pot	Apple sauce (Fe pot vs. non Fe pot vs. raw): 6.26mg vs. 0.18mg vs. 0.26mg/100g (p>0.05), Spaghetti sauce (Fe vs. non-Fe pot vs. raw): 2.10mg vs. 0.44mg vs. 0.22mg/100 g (p<0.05)

*Fe: Iron,

+Hb: Hemoglobin,

++RCT: Randomised control trial,

#Al: Aluminium,

**IDA: Iron deficiency anemia,

+++SF: Serum ferritin,

##M:F : Male: female ratio in study
